# Honey bees are the dominant diurnal pollinator of native milkweed in a large urban park

**DOI:** 10.1002/ece3.3394

**Published:** 2017-09-10

**Authors:** James Scott MacIvor, Adriano N. Roberto, Darwin S. Sodhi, Thomas M. Onuferko, Marc W. Cadotte

**Affiliations:** ^1^ Department of Biological Sciences University of Toronto Scarborough Toronto ON Canada; ^2^ Department of Ecology and Evolutionary Biology University of Toronto Toronto ON Canada; ^3^ Department of Biology York University Toronto ON Canada

**Keywords:** *Apis mellifera*, host shift, invasive plants, pollination, pollinia, urban

## Abstract

In eastern North America, the field milkweed, *Asclepias syriaca* L. (Asclepiadaceae), is used in planting schemes to promote biodiversity conservation for numerous insects including the endangered monarch butterfly, *Danaus plexippus* (Linnaeus) (Nymphalidae). Less is known about its pollinators, and especially in urban habitats where it is planted often despite being under increasing pressure from invasive plant species, such as the related milkweed, the dog‐strangling vine (DSV), *Vincetoxicum rossicum* (Kleopow) Barbar. (Asclepiadaceae). During the *A. syriaca* flowering period in July 2016, we surveyed bees in open habitats along a DSV invasion gradient and inspected 433 individuals of 25 bee species in 12 genera for pollinia: these were affixed to bees that visited *A. syriaca* for nectar and contain pollen packets that are vectored (e.g., transferred) between flowers. Of all bees sampled, pollinia were found only on the nonindigenous honeybee, *Apis mellifera* (43% of all bees identified), as well as one individual bumblebee, *Bombus impatiens* Cresson. Pollinia were recorded from 45.2% of all honeybees collected. We found no relationship between biomass of DSV and biomass of *A. syriaca* per site. There was a significant positive correlation between *A. syriaca* biomass and the number of pollinia, and the proportion vectored. No relationship with DSV biomass was detected for the number of pollinia collected by bees but the proportion of vectored pollinia declined with increasing DSV biomass. Although we find no evidence of DSV flowers attracting potential pollinators away from *A. syriaca* and other flowering plants, the impacts on native plant–pollinator mutualisms relate to its ability to outcompete native plants. As wild bees do not appear to visit DSV flowers, it could be altering the landscape to one which honeybees are more tolerant than native wild bees.

## 1 INTRODUCTION

Bees are essential pollinators, and their long‐term persistence, especially in human‐dominated landscapes, requires an abundance and diversity of flowers that serve as pollen and nectar sources (Kearns & Inouye, 1997; Müller et al., 2006). Bee populations are declining globally (Kerr et al., 2015; Potts et al., 2010), and subsequently, actions to support pollinators have increased substantially in habitats around the world and include a range of participants from government to researchers to citizens (Vanbergen, 2013). One plant often used in planting schemes aimed at supporting pollinator diversity in northeastern North America is the field milkweed, *Asclepias syriaca* L. (Asclepiadaceae). *Asclepias syriaca* is a native perennial herb and the oviposition site for the endangered monarch butterfly, *Danaus plexippus* Linnaeus (Nymphalidae), as well as a food plant for countless other insect species (Urquhart, 1960). Although once considered a “weed” in Ontario, it was removed from the noxious weed list in 2014 (Shahani, del Río Pesado, Schappert, Serrano, & Oberhauser, 2015).

**Figure 1:**
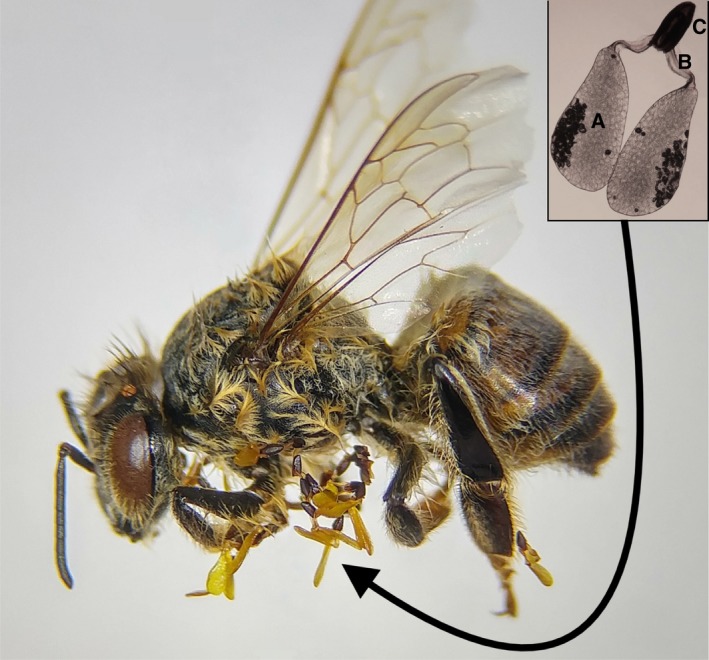
A honeybee affixed with numerous pollinia. Inset image: picture of *Asclepias syriaca* pollinium; (a) pollen packet, (b) translator arm, (c) corpusculum. Photograph credit: Marylouisse Feliciano

Milkweed flower morphology and pollination are unusual compared to other flowering plants (Wyatt, 1976). Within each flower, nectar is secreted inside five stigmatic chambers (Galil & Zeroni, 1965). The accumulating nectar attracts many pollinators; however, no pollen is consumed. Milkweed pollen is massed into packets onto structures called pollinia, which include two pollen masses (packets) joined via translator arms to a corpusculum (Figure 1). The corpuscula of each pollinium is set above one of the five stigmatic chambers and when a pollinator visits, it inadvertently removes the pollinia with their appendages when seeking nectar from a flower and deposits them in the same manner into different flowers (Fritz & Morse, 1981). When the pollinator visits a second milkweed flower, the pollen packets split off from the corpuscula, which remain on the pollinator and can be counted. Thus, vectored pollen loads and the proportions potentially transferred to flowers can be estimated from the number of “complete” pollinia and “deposited” pollinia (corpusculum and translator arms only) remaining on insect body parts (Kephart & Theiss, 2004; Robertson, 1929).

*Asclepias syriaca* has been shown to attract diverse floral “generalist” pollinators, including bumblebees (*Bombus s*pp.) and the nonindigenous Western Honey bee (*Apis mellifera* L.) (Ivey, Martinez, & Wyatt, 2003; Kephart & Theiss, 2004; Robertson, 1929). For example, Morse (1981) showed that the native *Bombus vagans* Smith and *Bombus terricola* Kirby made up 50.2% and 21.5% of all visits, respectively, and honeybees accounted for 12.3% of visits in undisturbed rural fields in Maine. In the same study region, bumblebees accounted for 44%–73% of *A. syriaca* pollination that resulted in mature seed pods, and in areas where nonindigenous honeybees were absent, this increased to as high as 95% (Morse, 1985).

Although urbanization and agriculture are known threats to milkweed populations (Brower et al., 2012; Oberhauser et al., 2001), the impact of invasive species is much less studied. Many North American habitats are experiencing unprecedented invasion by herbaceous vegetation that outcompetes key resource plants such as milkweed. For example, *Vincetoxicum rossicum* (Kleopow) Barbar. (Asclepiadaceae), known as the Dog‐strangling vine (DSV) in Canada, is a perennial vine native to Ukraine and Russia and has become very invasive in eastern North American open meadow and forest understory habitats, particularly between Ottawa and London in Ontario, Canada, and in parts of Michigan, Ohio, Pennsylvania, and New York states in the USA (DiTommaso, Lawlor, & Darbyshire, 2005; Ernst & Cappuccino, 2005; Sheeley & Raynal, 1996). Once established, DSV displaces most surrounding herbaceous vegetation indiscriminately through rapid growth that quickly overtakes and “strangles” surrounding plants, including tree saplings, as they compete for access to sunlight (Antunes & Sanderson, 2013). DSV is also classified as a milkweed (Bongard, Navaranjan, Yan, & Fulthorpe, 2013) and so the potential competitive interactions for habitat are amplified as DSV establishes and exploits niches similar to those occupied by *A. syriaca*.

In this study, we were interested in the potential direct and indirect impacts of DSV on the pollination of *A. syriaca* by bees, which can be inferred by the collection of pollinia on the bodies of bee specimens obtained in field surveys. DSV could directly impact *A. syriaca* abundance through competition for space and nutrients, and indirectly by impacting the number and type of pollinators visiting *A. syriaca*, thereby increasing pollen limitation and reducing seed set. We were interested in identifying the community of diurnal pollinia‐carrying bee visitors to *A. syriaca* and DSV flowers in our study area to address two hypotheses: (i) that greater DSV abundance will be negatively correlated with *A. syriaca* abundance, as DSV directly displaces native vegetation and (ii) that increasing DSV invasion will indirectly lead to lower rates of pollinia vectored between flowers of *A. syriaca*.

## 2 METHODS

### 2.1 Site

All sampling occurred in 2016 in the Rouge National Urban Park (http://www.pc.gc.ca/eng/pn-np/on/rouge/index.aspx) (total area: 79.1 km^2^), located in the northeast of Toronto, which is Canada's largest city (metro Toronto population: >5 million). The park has a multitude of different habitats and uses, including agricultural and industrial activity. While ecological restoration initiatives are ongoing throughout the park, there remain many heavily degraded habitats that include established populations of DSV and other invasive species. Honeybees are not feral in the park; they are managed in hives within the region due to agricultural activities in the region, but information on the locations or numbers of hives is not available. In the southern edge of the park within the boundaries of the city of Toronto, we sampled at seven open meadow sites experiencing different levels of DSV invasion. All sites were 50 × 50 m, at least 300 m apart, and with a ground layer of grasses, herbaceous flowers, and small shrubs.

### 2.2 Plant biomass

Each 50 × 50 m site was broken down into 1 × 1 m grid cells, and five 1 × 1 m cells (hereafter referred to as “plots”) were randomly selected for measuring plant biomass, which required complete above‐ground plant removal. In late June 2016, all individuals of DSV and *A. syriaca* per plot were identified, removed, and put in a standing drying oven at 60°C for a minimum of 48 hr until constant weight was reached. The dried samples were then weighed (in grams) to determine dry biomass per plot (g/m^2^). For each species, site‐level biomass was determined by taking the average biomass value from each of the five plots.

### 2.3 Bee sampling

Each site was surveyed for bees over three sampling dates (July 4, 11, and 19) during the flowering period of *A. syriaca* in July 2016. Two individuals simultaneously used hand nets to sample bees while foraging on clear, nonwindy days between 8:30 and 15:30 EDT. All sites were sampled on a single day for 30 min with each person sampling a minimum 15 m apart from one another. Collected bees were euthanized in a −21°C freezer at the end of each collection day. Bees were pinned, identified, and then inspected using a dissecting microscope for pollinia. The number of “complete” pollinia (with pollen packets remaining) and the number of “deposited” pollinia (with pollen packets transferred and only the corpusculum remaining) were counted (Figure 1), the latter indicating pollinia were vectored into another *A. syriaca* flower. Pollinia removal from flowers and insertion rates by insect pollinators are well correlated; therefore, comparing the number of pollinia with and without pollen packets remaining can provide a proxy for the proportion of pollinia vectored between flowers by visiting bees (Willson & Bertin, 1979; Wyatt, 1976). All bee voucher specimens are housed in the collection of the MacIvor Lab at the University of Toronto Scarborough in the Department of Biological Sciences.

### 2.4 Analysis

To examine relationships between the abundance of *A. syriaca* and DSV, we used a linear mixed effects model to compare biomass of each plant species, calculated from the five plot‐level samples from each of the seven sites (*N *=* *35) with site included as a random factor. We used linear regression analysis to examine the relationship between site‐level biomass of *A. syriaca* and biomass of DSV with wild (nonhoney) bee species richness and abundance from samples summed over all three dates (July 4, July 11, and July 19). Mixed effects models were used to examine the total number of “complete” pollinia (including pollen packets) and “deposited” pollinia (corpusculum only) collected by honeybees, and the proportion of pollinia vectored (calculated as “deposited” pollinia divided by the total; e.g., “complete” + ”deposited”) with site‐level *A. syriaca* biomass and DSV biomass. Site and the time (each 30‐min block) during which the site was sampled for bees were included as random factors in each model. Analyses were completed using the lmer and lmerTest package (Kuznetsova, Brockhoff, & Christensen, 2015) with the R statistical program v3.2.2 (R Core Team, 2015).

## 3 RESULTS

Over the three sampling periods during which *A. syriaca* was in bloom, hand netting yielded 433 bees from 25 species in 12 genera (Table S1). All bees collected were female. Of this total, 186 individuals were honeybees, and of these, 43.0% had at least one *A. syriaca “*complete” pollinium attached to an appendage (legs and/or mouthparts). On average, those honeybees with *A. syriaca* pollinia attached had 5.93 ± 2.01 on them, and the most recorded from a single honeybee was 23 (Figure 1). No DSV pollinia were identified on any of the bees collected in this study and pollinia from *A. syriaca* were observed on honeybees only, except for a single *Bombus impatiens* Cresson bearing two “deposited” pollinia (corpuscula only) caught on July 11th. The majority of honeybees (80.5%) having “complete” pollinia or corpusculum only attached were collected before 12:00 p.m. on each sampling day. There was no relationship between mean site‐level *A. syriaca* biomass and wild bee richness (*t*_4_ = −0.625, *p *=* *.566) or abundance (*t*_4_ = −0.652, *p *=* *.550) or mean site‐level DSV biomass and wild bee richness (*t*_4_ = 0.743, *p *=* *.499) or abundance (*t*_4_ = 1.176, *p *=* *.305).

**Figure 2:**
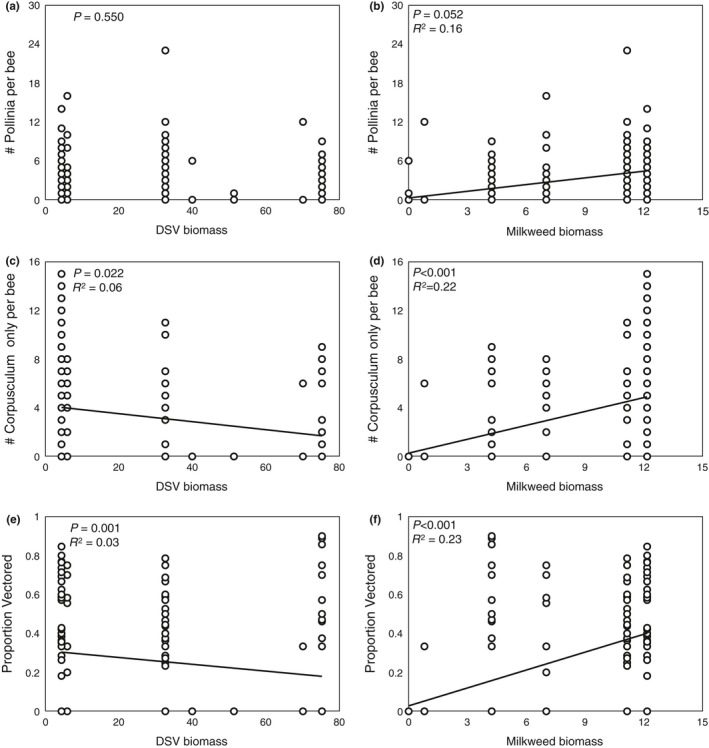
Relationship between biomass (g/m^2^) of *Vincetoxicum rossicum* (DSV) and *Asclepias syriaca* on the average number of pollinia per bee (a, b), corpusculum only per bee (c, d), and the proportion of pollinia vectored per bee (#of corpuscula only divided by the number of complete pollinia + corpuscula only) (e, f)

There was a negative but nonsignificant relationship between DSV biomass and *A. syriaca* biomass at the plot (*F*_1,33_ = 0.562, *p *=* *.459) and site level (*F*_1,5_ = 0.3.869, *p *=* *.106). DSV was found at 88.6% (*N *=* *31) of all plots surveyed and *A. syriaca* in 48.6% of sites (Table 1). There was a marginally significant positive relationship between the biomass of *A. syriaca* per site and the number of “complete” pollinia on honeybee individuals (*t*_159_ = 2.748, *p *=* *.052; Figure 2b), as well as a significant relationship with the number of corpusculum only that were attached per honeybee (*t*_159_ = 5.190, *p *<* *.001; Figure 2d), the proportion of pollinia vectored (*t*_159_ = 6.047, *p *<* *.001) (Figure 2f), and the total (pollinia + corpusculum only) (*t*_159_ = 4.695, *p *=* *.116). A significantly negative relationship between increasing DSV biomass and the proportion of corpusculum per honeybee (*t*_159_ = 2.314, *p *=* *.022) (Figure 2c), as well as between the proportion of pollinia vectored (*t*_159_ = 3.334, *p *=* *.001) (Figure 2e) was determined, but not the number of “complete” pollinia (*t*_159_ = 0.676, *p *=* *.550) (Figure 2a) or the total (pollinia + corpusculum only) (*t*_159_ = 1.595, *p *=* *.231).

**Table 1:** Site‐level summary of bees collected, average, and total complete *Asclepias syriaca* pollinia and corpusculum (mean ± *SE*) and the proportion vectored, as well as the biomass of *A. syriaca* and DSV

Site	Coordinates	Complete pollinia		Corpusculum only		Proportion vectored	# of bees with *A. syriaca* pollinia	*A. syriaca* (g/m^2^)	DSV (g/m^2^)
		Mean	Total	Mean	Total				
I	43.840040, −79.203549	3.86 ± 0.56	170	4.93 ± 0.67	217	0.37 ± 0.05	34	12.17	4.35
II	43.839996, −79.204556	2.22 ± 0.85	51	2.26 ± 0.65	52	0.14 ± 0.05	9	7.03	5.92
III	43.836651, −79.194899	5.34 ± 0.85	171	4.69 ± 0.52	150	0.44 ± 0.04	26	11.15	32.62
IV	43.823127, −79.155829	0.30 ± 0.30	6	0 ± 0	0	0	1	0	39.95
V	43.811403, −79.162095	0.25 ± 0.25	1	0 ± 0	0	0	1	0	51.31
VI	43.814847, −79.167009	3.00 ± 3.00	12	3.00 ± 1.73	12	0.08 ± 0.08	1	0.80	70.09
VII	43.837385, −79.192242	1.34 ± 0.42	43	2.06 ± 0.56	66	0.22 ± 0.06	12	4.24	75.27

## 4 DISCUSSION

In a large urban park, we found that >99% of diurnal pollination of the field milkweed, *A. syriaca* was carried out by the nonindigenous Western Honey bee, *Apis mellifera*. Our findings suggest that *A. syriaca* is pollinated predominantly by honeybees in human‐impacted region, unlike in similar studies carried out in natural areas decades earlier in which bumblebees were the dominant visitor (e.g., Macior, 1965; Morse, 1985). In this study, 55% of all bees sampled were wild (nonhoney) bees, but none had pollinia attached. Many of these bee species are presumably small enough to obtain nectar while avoiding the pollinia, while others might avoid *A. syriaca* to minimize interactions with honeybees. *Asclepias syriaca* does however provide significant habitat for numerous other important insects, including the endangered monarch butterfly, which uses the plant for oviposition (Inamine, Ellner, Springer, & Agrawal, 2016). Moreover, bumblebees are known visitors to *A. syriaca* but are experiencing declines in North America (Cameron et al., 2011; Kerr et al., 2015), and so for these purposes should be promoted in planting initiatives around metropolitan Toronto and throughout the region.

### 4.1 Impacts of invasive DSV on *Asclepias syriaca* biomass

We found a negative but nonsignificant relationship between increasing biomass of DSV and *A. syriaca* biomass at the plot‐ and site level and do not have sufficient evidence to accept our first hypothesis that the invasive plant has a negative impact on the presence of *A. syriaca*. Both of these plants are milkweeds and so likely overlap in their fundamental niche space leading to high competition between these species, although our data do not display evidence of such negative interaction. DSV has been shown to have negative impacts on an assortment of taxa. For example, Ernst and Cappuccino (2005) found that DSV supported the lowest number of phytophagous insects, as well as stem‐ and ground‐dwelling arthropods, and supports almost no pollinators.

We found a negative but nonsignificant effect of DSV biomass on the number of pollinia affixed to bees in our study system. As we did not find any DSV pollinia on any bees in this study, these findings support previous observations that little to no insects are attracted to DSV flowers (St. Denis & Cappuccino, 2004); however, Lumer and Yost (1995) noted 14 species of fly visiting the related *Vincetoxicum nigrum*, which is also introduced to northeastern North America and occurs in similar habitats (Gleason & Cronquist, 1991). The pollinia of DSV are much smaller and easily distinguishable from that of the related *A. syriaca*. Due to observations of ants on DSV flowers and stems (J. S. M. and M. W. C., personal observation), it is likely that ants are the most obvious visitor to DSV for nectar in our region. As a result, DSV is not likely impacting *A. syriaca* indirectly by being more attractive to pollinators; rather, DSV's mode of successful invasion is a function of its growth morphology and ability to bind up and overgrow all surrounding vegetation, allowing it greater access to sunlight. This highlights the importance of partitioning the different direct and indirect impacts that invasive species can have on communities for determining management and biocontrol strategies.

### 4.2 Pollinia as an indicator of milkweed pollinators versus milkweed visitors

We found that more than half of all bees identified in this study did not have pollinia attached—including all of the nonmanaged wild bees—despite the milkweed *A. syriaca* providing rich and abundant nectar as an attractant to pollinators. Pollinia are big structures (Figure 1) compared to individual pollen grains, and we found that only honeybees and one bumblebee were identified as *A. syriaca* pollinators in our study. Other bee species identified (Table S1) could include *A. syriaca* visitors seeking nectar but are too small and not nearly as hairy as compared to honeybees and bumblebees for pollinia attachment. More work is needed to identify and differentiate bee pollinators from bee visitors of *A. syriaca*, as the plant may have great value in pollinator management planning as a nectar resource.

### 4.3 Honeybees as primary pollinators of native *Asclepias syriaca*

Honeybees have been present in North America for centuries and are common visitors to *A. syriaca*. In our study, we found that the introduced honeybee is the primary pollinator of the native *A. syriaca*. This finding has been repeated in other recent studies of the plant; for example, in a grassland remnant in the city of Bloomington, Indiana, Theiss, Kephart, and Ivey (2007) found that honeybees visited *A. syriaca* the most and foraged on it longer than native bee species. Also, in disturbed and remnant areas near Urbana, Illinois, Willson and Bertin (1979) found that honeybees accounted for 26%–49% of vectored *A. syriaca* pollinia and were the most abundant pollinator of *A. syriaca*. By contrast, findings from studies over 30 years ago indicate that native bumblebees, not honeybees, were the primary pollinators of *A. syriaca*. For example, in wet mesic‐ and dry prairie habitats, Macior (1965) noted the native *Bombus griseocollis* (De Geer) was the primary pollinator of *A. syriaca*. In rural Bremen, Maine, Fritz and Morse (1981) found 97% of bumblebees recorded had pollinia affixed to them compared to only 9% of honeybees. Further, in Morse (1982), the author describes more than 88% of bumblebees recorded having more than one pollinia affixed. It is possible that honeybees visit *A. syriaca* in higher proportions in and around urban areas where they are managed more abundantly, but more work is needed.

Although these findings illustrate the role of honeybees as pollinators of native plants (Aizen, Morales, & Morales, 2008), the high number of honeybees found with pollinia affixed and the complete lack of other diurnally active pollinator species could reflect interference competition (Huryn, 1997; Schaffer et al., 1983), or strong selection for *A. syriaca* to attract honeybees over bumblebee species. Our evidence indicated that most honeybee visits to *A. syriaca* (80.5%) occur before 12:00 p.m. Willson and Bertin (1979) have suggested that honeybees have a selective pressure on *A. syriaca* floral biology such that the timing of nectar availability and peak flowering is more aligned with honeybee activity instead of that of other native pollinators. For example, they found that the abundance of honeybee visitors to *A. syriaca* coincided with a shift in the temporal patterns of total pollinator visits (Willson & Bertin, 1979). Many visitors to *A. syriaca* are nocturnal, and the authors found a shift in nectar production to earlier in the day, when honeybees are foraging, from previously typical production in early evening for attracting nocturnal moths. Honeybees are abundant and good dispersers and thus could bring higher quantities of pollen to *A. syriaca*, thereby replacing some of the services that native pollinators provide to *A. syriaca* plants.

With populations of bumblebees declining across North America (Kerr et al., 2015), the importance of managed honeybees could become increasingly important as pollinators of *A. syriaca*, especially for species that depend on the plant for nesting, such as the Monarch butterfly. Milkweed requires nonselfed pollinia transfer for reproductive success, and in natural areas they can be pollinia‐limited. For example, Morse & Fritz (1983) determined that over two different study years, only 25%–50% of *A. syriaca* pods matured compared to what would be expected had there been adequate nonselfed pollinia provided by pollinators. Prioritization of wild bees such as bumblebees to sustain native plants in natural areas is thus essential when evaluating plans for conservation of plant–pollinator mutualisms and diverse pollination networks. Promoting honeybees to pollinate native milkweed will also lead to potentially lessened pollination service as many of the pollinia are wasted because pollen packets detach and accumulate at the entrance to honeybee hives (Willson & Bertin, 1979). Nevertheless, Identifying honeybees as primary pollinators of *A. syriaca* where bumblebees are less abundant indicates potential short‐term solutions to ensure these plants are effectively pollinated to support other native insect species of conservation concern (e.g., Monarch butterflies). However, this strategy could have unintended consequences for wild bee diversity through enhanced competition for other (nonmilkweed) flowering plants (Cane & Tepedino, 2017).

## AUTHOR CONTRIBUTIONS

JSM and MWC conceived the study. JSM and AR collected bee and pollinia data. TMO, JSM, and AR identified bees to species. DS collected plant data. JSM conducted the analysis. All authors contributed to the writing of the manuscript.

## Supporting information


** **
