# Supplementary material for: Honey bees are the dominant diurnal pollinator of native milkweed in a large urban park
Source: Ecol Evol. 2017 Sep 10;7(20):8456–62. doi: 10.1002/ece3.3394 (PMC5648680; doi:10.1002/ece3.3394)
Supplement: Supplementary file 1 [file ECE3-7-8456-s001.docx]

|  |  | Site | | | | | | | |  |
| --- | --- | --- | --- | --- | --- | --- | --- | --- | --- | --- |
| Genus/Species Name | **Family** | **I** | **II** | **III** | **IV** | **V** | **VI** | **VII** | **VIII** | **Total** |
| *Andrena nivalis* Smith | Andrenidae | 2 | 0 | 0 | 0 | 0 | 0 | 0 | 0 | 2 |
| *Andrena wilkella* (Kirby) | Andrenidae | 2 | 1 | 2 | 2 | 4 | 0 | 0 | 0 | 11 |
| *Apis mellifera* Linnaeus | Apidae | 44 | 23 | 32 | 20 | 4 | 4 | 32 | 27 | 186 |
| *Bombus bimaculatus* Cresson | Apidae | 2 | 2 | 0 | 4 | 3 | 14 | 3 | 2 | 30 |
| *Bombus borealis* Kirby | Apidae | 1 | 0 | 0 | 0 | 0 | 0 | 0 | 0 | 1 |
| *Bombus fervidus* (Fabricius) | Apidae | 0 | 0 | 1 | 0 | 0 | 2 | 0 | 0 | 3 |
| *Bombus griseocollis* (De Geer) | Apidae | 0 | 0 | 0 | 0 | 9 | 2 | 0 | 0 | 11 |
| *Bombus impatiens* Cresson | Apidae | 0 | 0 | 0 | 0 | 1 | 0 | 0 | 0 | 1 |
| *Bombus rufocinctus* Cresson | Apidae | 0 | 2 | 15 | 5 | 2 | 0 | 0 | 0 | 24 |
| *Ceratina calcarata* Robertson | Apidae | 0 | 0 | 0 | 0 | 0 | 1 | 0 | 1 | 2 |
| *Ceratina dupla* Say | Apidae | 1 | 0 | 0 | 0 | 0 | 0 | 0 | 1 | 2 |
| *Melissodes druriella* (Kirby) | Apidae | 0 | 0 | 0 | 0 | 0 | 0 | 1 | 0 | 1 |
| *Melissodes illata* Lovell and Cockerell | Apidae | 0 | 0 | 0 | 0 | 3 | 7 | 0 | 0 | 10 |
| *Nomada articulata* Smith | Apidae | 0 | 0 | 0 | 0 | 0 | 1 | 0 | 0 | 1 |
| *Hylaeus affinis* (Smith) | Colletidae | 0 | 0 | 0 | 0 | 0 | 1 | 0 | 0 | 1 |
| *Dufourea monardae* (Viereck) | Halictidae | 0 | 0 | 0 | 0 | 2 | 9 | 0 | 0 | 11 |
| *Halictus confusus* Smith | Halictidae | 0 | 2 | 0 | 2 | 2 | 4 | 0 | 0 | 10 |
| *Halictus ligatus* Say | Halictidae | 0 | 8 | 1 | 13 | 2 | 25 | 16 | 2 | 92 |
| *Halictus rubicundus* (Christ) | Halictidae | 4 | 0 | 2 | 0 | 2 | 0 | 0 | 0 | 8 |
| *Lasioglossum anomalum* (Robertson) | Halictidae | 0 | 0 | 1 | 0 | 0 | 2 | 0 | 1 | 4 |
| *Lasioglossum laevissimum* (Smith) | Halictidae | 0 | 1 | 1 | 0 | 0 | 0 | 1 | 0 | 3 |
| *Lasioglossum perpunctatum* (Ellis) | Halictidae | 0 | 2 | 0 | 0 | 0 | 1 | 0 | 0 | 3 |
| *Lassioglossum versatum* (Robertson) | Halictidae | 0 | 1 | 4 | 0 | 0 | 0 | 2 | 0 | 7 |
| *Megachile pugnata* Say | Megachilidae | 0 | 0 | 0 | 0 | 6 | 0 | 0 | 0 | 6 |
| *Hoplitis pilosifrons* (Cresson) | Megachilidae | 0 | 0 | 1 | 0 | 0 | 0 | 0 | 2 | 3 |
|  | |  |  |  |  |  |  |  |  |  |
| Total | | 42 | 56 | 60 | 55 | 46 | 40 | 73 | 36 | 433 |

Supplemental table 1. List of bee species collected during the study of milkweed pollination. Site VIII was removed from analysis because no plant biomass data were available.
